# Marker-Based Estimation of Genetic Parameters in Genomics

**DOI:** 10.1371/journal.pone.0102715

**Published:** 2014-07-15

**Authors:** Zhiqiu Hu, Rong-Cai Yang

**Affiliations:** 1 Department of Agricultural, Food and Nutritional Science, University of Alberta, Edmonton, Alberta, Canada; 2 Alberta Agriculture and Rural Development, Edmonton, Alberta, Canada; University of Miami, United States of America

## Abstract

Linear mixed model (LMM) analysis has been recently used extensively for estimating additive genetic variances and narrow-sense heritability in many genomic studies. While the LMM analysis is computationally less intensive than the Bayesian algorithms, it remains infeasible for large-scale genomic data sets. In this paper, we advocate the use of a statistical procedure known as symmetric differences squared (SDS) as it may serve as a viable alternative when the LMM methods have difficulty or fail to work with large datasets. The SDS procedure is a general and computationally simple method based only on the least squares regression analysis. We carry out computer simulations and empirical analyses to compare the SDS procedure with two commonly used LMM-based procedures. Our results show that the SDS method is not as good as the LMM methods for small data sets, but it becomes progressively better and can match well with the precision of estimation by the LMM methods for data sets with large sample sizes. Its major advantage is that with larger and larger samples, it continues to work with the increasing precision of estimation while the commonly used LMM methods are no longer able to work under our current typical computing capacity. Thus, these results suggest that the SDS method can serve as a viable alternative particularly when analyzing ‘big’ genomic data sets.

## Introduction

Recent surge of genome-wide association studies (GWAS) based largely on the use of single nucleotide polymorphisms (SNPs) has enabled plant/animal breeders and human geneticists to identify hundreds of SNPs responsible for the genetic variation of quantitative traits or complex diseases. While such identification contributes to an in-depth understanding of the genetic architecture of complex traits, the numerous SNPs identified have collectively accounted for only a small amount of genetic variation in many studies. Several possible explanations have been put forward to explain this phenomenon known as the “missing heritability” [1]–[4]. Meanwhile, several studies [5]–[8] suggested the use of Henderson’s [9] linear mixed models (LMM) to estimate the total genetic variation captured by all SNPs by replacing the pedigree-based relationship matrix with the marker-based relationship matrix in the mixed-model equations. As usual, restricted maximum likelihood (REML) method is used for the estimation of variance components. The use of LMM uncovers a substantial amount of hidden (rather than missing) heritability. However, the LMM-REML analysis is computationally very demanding particularly when there are a large number of individuals. For this reason, there are now a whole array of software packages including ASREML [10], EMMA [6], [11], FaST-LMM [12], GEMMA [8], GCTA [13], rrBLUP [14] and TASSEL [15] that implement the REML in the LMM analysis.

Since Meuwissen et al. [16], the use of DNA markers over the whole genome for prediction of unobserved phenotypes (genome-wide prediction or genomic selection, GS) has been extensively exploited in animal and plant breeding [17], [18]. While the Bayesian-based analyses are often used to simultaneously estimate variance parameters and marker effects in GS studies, it is recommended [17] that with moderate to large sample sizes, a two-step approach should be employed: (i) to estimate the variance components using a non-Bayesian algorithm, usually REML-based algorithm; and (ii) to compute BLUP of genetic effects from standard mixed-model equations. This two-step approach is computationally less intensive than the Bayesian algorithms.

In order to achieve sufficient statistical power of detecting numerous variants with small-sized effects that collectively contribute to the genetics of most complex traits, large sample sizes are often used [19]–[23] though it is most convenient to scan the whole genome one marker at a time. For example, a recent large-scale (126,559 individuals) GWAS [23] is able to detect very small allelic effect with *R* ^2^ of ∼ 0.02%. However, the LMM analysis of such large data sets creates a much heavier computational burden because the computing time required for constructing the genetic relationship matrix and solving LMM equations increases with the cube of the number of individuals fit as a random effect. The computing time is further increased because iteration is needed to estimate population parameters, such as variance components that are due to the effects of individual tested markers. Despite recent proposed improvements [e.g., 8,12], the computational burden with the LMM analysis will remain to be a major issue in anticipation of larger GWAS or GS studies in the future.

It has been well known since the founding days of quantitative genetics [24] that genetic and environmental variance components can be estimated directly from phenotypic resemblance between related individuals. Koch [25] proposed the use of symmetric differences squared (SDS) as a general statistical procedure to compute the phenotypic resemblance between relatives. The SDS procedure or its variant based on the phenotypic similarity index have been subsequently used to estimate genetic and environmental variances for linkage analysis [24], animal breeding [25], estimation of heritability in natural populations [26]–[29], and recently in the genetic analysis of complex traits in humans [3], [5], [30] and QTL mapping in plants [31], [32]. Since the SDS procedure is a general and computationally simple method based only on the least squares regression analysis, it may serve as a viable alternative when the REML-based methods have difficulty or fail to work with large datasets. However, the SDS procedure has not always been employed appropriately and its correct usage needs to be clarified. The objectives of this study are (i) to provide a comprehensive evaluation of the SDS procedure in terms of its statistical properties and computational efficiencies and (ii) to compare it with two commonly used REML-based methods implemented in the software packages, rrBLUP and GCTA.

## Results

### Simulation results

**Figure 1:**
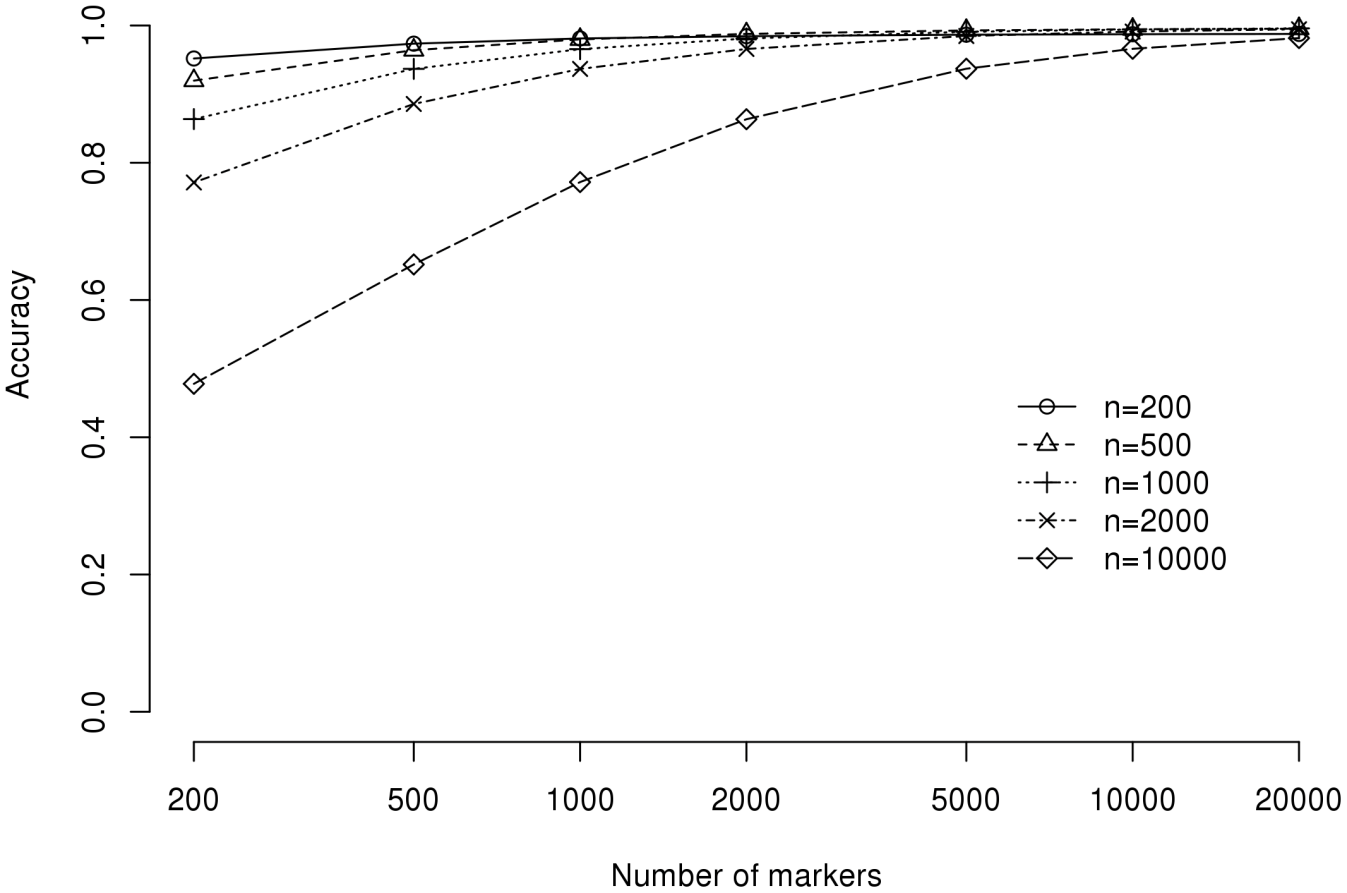
Effects of marker density on correlation between realized genetic relatedness and its expected value under the AR1 model.

The effects of sample size and marker density on the correlation between the actual and theoretical genetic relatedness are depicted in Figure 1. The correlations are nearly perfect (*r* >0.98) with the high marker density (*m* = 20000) regardless of sample sizes. However, the situation is different for lower marker densities: while there is little change in the correlations for smaller samples, the correlations for larger samples can be substantially reduced. For example, at the marker density of *m* = 200, the correlation for the population of size *n* = 200 is *r* = 0.95, but the correlation for the sample of size *n* = 10000 is nearly halved at *r* = 0.48.

Presented in Table 1 are the means and ranges of realized heritability values (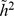) and SDS and REML heritability estimates (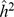) from 100 replicated samples for each of the 27 simulation combinations consisting of theoretical heritability, sample size and marker density. A realized heritability is calculated as the ratio of the variance components directly from simulated genetic and residual effects and thus it gauges the variation in genetic sampling across different simulated samples. The effect of genetic sampling is evident as the 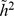 values are distributed around their respective true values (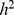). Such effect is more evident with smaller samples but the impact of marker density is less obvious. Since the estimates by rrBLUP and GCTA are identical, the mean values and ranges of estimated heritability under the header of REML are those of GCTA estimates. Both REML and SDS approaches give the mean values that are close to the corresponding true values of heritability for all the parameter combinations. The *t*-tests show that with a few exceptions (seven in the REML estimates and one in the SDS estimates), the means of the estimates are not significantly different from their theoretical values. However, while the ranges of the estimated heritability by the REML approach are relatively consistent over different sample sizes and marker densities, the ranges by the SDS approach are much larger for *n* = 500 than for *n* = 5000 under different *h* ^2^ and *m* values. For example, for the parameter combination of *h* ^2^ = 0.8 and *m* = 200, the ranges of REML estimates are 0.075 ( = 0.828–0.753) for *n* = 500 and 0.014 (0.806–0.792) for *n* = 5000, but the ranges of SDS estimates are 0.558 (1.096–0.538) for *n* = 500 and 0.268 (0.955–0.687) for *n* = 5000. Unlike the sample size, the marker density has a minor effect on the estimates of heritability though there are larger standard deviations and ranges of heritability estimates when too many markers (*m* = 20000) are used.

**Table 1:** Means and 90% ranges of 100 SDS and REML estimates of narrow-sense heritability (*h* ^2^) for 27 simulation trials^a^.

			Realized 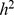 ( 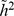 )		SDS		REML	
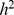	n	m	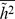 ± SD	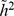 Range of	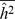 ± SD^b^	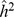 Range of	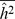 ± SD	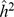 Range of
0.2	500	200	0.187±0.030	0.146–0.243	0.193±0.078	0.081–0.313	0.187±0.047*	0.121–0.249
0.2	500	2000	0.190±0.032	0.140–0.244	0.197±0.077	0.082–0.342	0.199±0.065	0.095–0.303
0.2	500	20000	0.186±0.031	0.136–0.238	0.184±0.088	0.054–0.319	0.192±0.069	0.095–0.300
0.2	1000	200	0.194±0.029	0.153–0.243	0.198±0.069	0.106–0.337	0.197±0.039	0.134–0.261
0.2	1000	2000	0.189±0.022	0.157–0.223	0.192±0.045	0.118–0.262	0.193±0.040	0.132–0.263
0.2	1000	20000	0.191±0.024	0.155–0.228	0.201±0.086	0.091–0.342	0.194±0.048	0.113–0.273
0.2	5000	200	0.199±0.011	0.181–0.216	0.196±0.041	0.136–0.275	0.198±0.011	0.179–0.216
0.2	5000	2000	0.199±0.013	0.181–0.218	0.198±0.039	0.149–0.269	0.199±0.016	0.173–0.223
0.2	5000	20000	0.200±0.011	0.184–0.217	0.204±0.050	0.124–0.297	0.201±0.019	0.174–0.233
0.5	500	200	0.476±0.049	0.407–0.562	0.492±0.138	0.296–0.725	0.482±0.048*	0.415–0.561
0.5	500	2000	0.480±0.052	0.394–0.564	0.495±0.119	0.300–0.702	0.499±0.070	0.384–0.602
0.5	500	20000	0.474±0.051	0.386–0.556	0.480±0.150	0.271–0.694	0.480±0.085*	0.337–0.602
0.5	1000	200	0.487±0.046	0.419–0.562	0.494±0.114	0.344–0.683	0.495±0.035	0.435–0.548
0.5	1000	2000	0.481±0.036	0.428–0.535	0.482±0.072*	0.374–0.613	0.491±0.047	0.417–0.565
0.5	1000	20000	0.485±0.038	0.423–0.542	0.511±0.144	0.343–0.774	0.490±0.050	0.408–0.563
0.5	5000	200	0.498±0.017	0.470–0.525	0.497±0.063	0.408–0.615	0.497±0.010*	0.479–0.513
0.5	5000	2000	0.497±0.020	0.468–0.528	0.498±0.052	0.428–0.595	0.498±0.016	0.472–0.521
0.5	5000	20000	0.499±0.017	0.474–0.526	0.507±0.069	0.411–0.609	0.501±0.022	0.464–0.534
0.8	500	200	0.782±0.034	0.733–0.837	0.802±0.177	0.538–1.096	0.790±0.022*	0.753–0.828
0.8	500	2000	0.785±0.035	0.722–0.838	0.801±0.135	0.592–1.022	0.797±0.044	0.725–0.860
0.8	500	20000	0.781±0.035	0.715–0.833	0.797±0.202	0.536–1.114	0.783±0.064*	0.670–0.874
0.8	1000	200	0.790±0.031	0.742–0.837	0.797±0.137	0.612–1.008	0.797±0.014	0.775–0.819
0.8	1000	2000	0.786±0.024	0.749–0.821	0.785±0.084	0.652–0.953	0.795±0.027	0.754–0.842
0.8	1000	20000	0.789±0.026	0.746–0.825	0.829±0.191	0.573–1.189	0.796±0.032	0.736–0.841
0.8	5000	200	0.799±0.011	0.780–0.815	0.799±0.081	0.687–0.955	0.798±0.004*	0.792–0.806
0.8	5000	2000	0.798±0.013	0.779–0.817	0.800±0.057	0.715–0.899	0.799±0.007	0.787–0.809
0.8	5000	20000	0.799±0.011	0.783–0.816	0.809±0.088	0.665–0.937	0.801±0.013	0.778–0.819

*Indicates a significant deviation from *h* ^2^ according to a t-test: 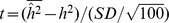.

a The 27 simulation trials consist of three levels of true narrow-sense heritability (*h* ^2^), three sample sizes (*n*) and three marker densities (*m*). In each simulation sample, *h* ^2^ is estimated by the symmetric difference squared (SDS) method implemented in our R package, SDS/R and by residual maximum likelihood (REML) method implemented in the GCTA software.

b SD = standard deviation.

The computational time and memory requirements for the two REML-based analyses (rrBLUP and GCTA) and the SDS analysis are recorded in Table 2 for seven sample sizes from *n* = 500 to *n* = 40000. These records of time and memory requirements are taken from the runs of the analyses on Dell Precision T1650 with Intel Xeon E3-1280 (3.8 GHz) and 32GB of RAM under the Redhat Linux 6.4 operating system. In anticipation of insufficient RAMs required for the REML-based analyses of large data sets, we decide that any analysis is terminated if its RAM usage exceeds 30GB. Thus, the recorded memory for a given analysis is the maximum memory usage over the entire process of the analysis if the memory usage at any given time of the analysis is within 30GB RAMs, or the maximum memory usage during the time period from the beginning to the termination of the analysis if the memory usage is beyond 30GB RAMs. In this study, the time period for terminating an analysis is set to be one hour after the 30GB RAM usage criterion is reached. For GCTA, only one CPU core is used in each analysis run to measure the computational time and memory usage for a fair comparison with the computational efficiencies for rrBLUP and SDS. For rrBLUP, the memory usage is measured in a clean R environment with only the 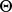 matrix and the vector of phenotypic values being loaded and the time of loading the data is not included in the computational time recorded.

**Table 2:** Actual computational efficiency by SDS and REML procedures under samples of seven sizes (*n*)^a^.

	REML					
	GCTA		rrBLUP		SDS	
n	Time (s)	RAM(GB)	Time (s)	RAM(GB)	Time (s)	RAM(GB)
500	0.235	<0.01	1.438	0.27	0.054	0.26
1000	0.746	0.02	17.856	0.42	0.061	0.32
2000	3.881	0.33	112.780	0.94	0.084	0.42
5000	58.451	1.91	2738.159	4.77	0.231	0.59
10000	226.827	7.46	9054.193	19.33	0.756	1.14
20000	1610.518	28.90	NA^b^	65.60	2.851	2.05
40000	NA	NA	NA	NA	11.286	5.13

a The computational times in seconds (s) and memory requirements in gigabites (GB) that are required to run a simulated sample of size n with the true heritability of *h* ^2^ = 0.5 and marker density of *m* = 2000 by the symmetric difference squared (SDS) and two residual maximum likelihood (REML) methods, GCTA and rrBLUP. Each of these times and memory requirements is an average over five simulation samples.

b NA indicates that no information is available due to termination of the analysis.

It is evident from Table 2 that the SDS analysis succeeds with all sample sizes and it requires far less computational time and memories than either the GCTA or rrBLUP analysis. For the two REML-based methods, while the rrBLUP and GCTA analyses give identical estimates of variance components and heritability, the GCTA analysis requires much less computational time and memories than the rrBLUP analysis. When the sample size is increased to *n* = 20000, the rrBLUP analysis is terminated because its memory requirement (65.6GB) is far beyond our criterion of 30GB RAMs. With this same sample size, the GCTA analysis is successfully completed in ∼27 minutes but its memory requirement (28.9GB) is near the RAM capacity of our computer. Obviously, both rrBLUP and GCTA are terminated for *n* = 40000 because the memory requirement certainly exceeds our criterion of 30GB RAMs.

### Empirical study

#### Wheat data

We will analyse two empirical examples. The first empirical data set is taken from Crossa et al. [33] and it includes 599 wheat inbred lines developed by the CIMMYT Global Wheat Breeding program and tested in four target sets of environments (E1–E4). The pedigree data and 1279 polymorphic Diversity Array Technology (DArT) markers for these lines are provided as well. Grain yield (GY) was measured in all the environments. Crossa et al. [33] compared the estimates of variance components and heritability under different schemes ranging from pedigree-based information only to the combination of both pedigree-based and marker information. Since the genotype of each inbred line can only be one of the two homozygotes at a marker locus (essentially a haploid model), the *il*th element of **W** matrix for the genotype of the *i*th individual at *l*th locus is coded as, 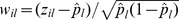, where the indicator variable *z_il_* is 1 for the reference homozygote and 0 for the alternative homozygote, and 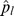 is the estimated frequency of the reference homozygote at the *l*th locus. Both rrBLUP and GCTA do not allow for inputting haploid data for constructing **W** matrix and thus the GRM, but GCTA does have an option to allow for inputting the GRM. Thus the comparison will be between the SDS and GCTA analyses for this empirical data.

The estimates of heritability for the GY in E1-E4 are presented in Table 3. The SDS estimates are comparable with the GCTA estimates, judging from the standard deviations and the 95% confidence intervals generated by bootstrapping. The bootstrapping procedure used here is somewhat different from the usual bootstrapping in that any duplicates in a bootstrap sample from sampling with replacement are excluded to avoid the problem of the GRM being singular in the REML-based GCTA estimation.

**Table 3:** SDS and REML estimates of heritability for wheat grain yield in four environments (E1–E4)^a^.

Environment	SDS			REML		
	*h* ^2^	SD	CI95 b	*h* ^2^	SD	CI95
E1	0.564	0.086	0.362–0.706	0.498	0.049	0.399–0.589
E2	0.452	0.114	0.192–0.629	0.448	0.048	0.324–0.520
E3	0.379	0.070	0.223–0.497	0.423	0.061	0.305–0.544
E4	0.481	0.071	0.304–0.587	0.430	0.058	0.292–0.524

a The estimates of heritability for the wheat data set taken from Crossa et al. [33] by the symmetric difference squared (SDS) method and a residual maximum likelihood (REML) method, GCTA.

b The 95% confidence intervals (CI 95) are constructed based on 1000 bootstrap samples.

#### Drosophila data

The second empirical data set is taken from Macdonald et al. [34]. These authors examined associations of 203 SNPs at the *Enhancer of split* Complex [(E(spl)-C] with sternopleural bristle number (SBN) and abdominal bristle number (ABN) for 2000 *Drosophila melanogaster* individuals (1000 males and 1000 females) sampled from a location in Napa Valley, California. The 203 genotyped polymorphisms actually consisted of 191 SNPs and 12 insertion/deletion events, but for simplicity, they were all referred to as SNPs. There was little evidence of associations between individual SNPs and SBN or ABN in the female or male population. In fact, Macdonald et al. [34] concluded that individual SNPs in the E(spl)-C gene region contributed little to phenotypic variation in SBN and ABN. For this reason, we use the LMM-REML and SDS methods to determine if the joint effect of all SNPs in the E(spl)-C would make a detectable contribution to the variation in SBN and ABN. Prior to our analysis, we remove 50 individuals with missing phenotypic values or genotype scores in both female and male populations. In addition, a total of 41 SNPs are removed due to (i) minor allele frequency (MAF) of <0.05 and (ii) significant (*p*<0.01) departure from Hardy-Weinberg proportions of three genotypes at each locus. Thus, 950 individuals with 162 SNPs in the E(spl)-C are retained for the analysis. To be consistent with the analysis of the first data set, only the GCTA is used for comparison with the SDS.

As shown in Table 4, the joint effect of 162 SNPs in the E(spl)-C makes a small contribution to the phenotypic variation in SBN and ABN in both female and male populations. The percentages of the contribution range from 0.5% to 9% by the SDS method and 0% to 1.4% by the GCTA. These are reasonable values given that (i) this is the joint contribution of potential causal variants from one major gene complex for the traits; and (ii) the previous gene-wide scan by Macdonald et al. [34] revealed only marginally significant associations of three SNPs with SBN and of two SNPs with ABN regardless of sexes. The lower limits of the 95% confidence intervals are all bound to 0 because, in a REML-based analysis, a constraint of 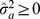 and thus 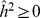 is imposed.

**Table 4:** SDS and REML estimates of heritability for bristle number in a large wild-caught cohort of fruit fly (*Drosophila melanogaster)* a.

		SDS			REML		
Sex	Trait^b^	*h* ^2^	SD	95% CI^c^	*h* ^2^	SD	95% CI
Female	ABN	0.035	0.027	−0.028–0.074	0.000	0.004	0.000–0.015
Female	SBN	0.008	0.023	−0.034–0.053	0.000	0.006	0.000–0.020
Male	ABN	0.090	0.039	−0.015–0.136	0.014	0.013	0.000–0.043
Male	SBN	0.005	0.027	−0.033–0.072	0.000	0.009	0.000–0.031

a The estimates of heritability for the fruit fly data set taken from Macdonald et al. [34] by the symmetric difference squared (SDS) method and a residual maximum likelihood (REML) method, GCTA.

b ABN = abdominal bristle number and SBN = sternopleural bristle number.

c The 95% confidence intervals (CI 95) are constructed based on 1000 bootstrap samples.

## Discussion

The computational burdens of many LMM applications including heritability estimation in large-scale genomic studies have recently stimulated a huge amount of research interest in the development of faster and memory-efficient algorithms for feasible and successful analyses of large-scale genomic data. Despite these efforts, the computational challenges remain. In this study, we investigate the statistical properties and computational efficiency of the least-squares-based SDS method in comparison to the two LMM methods (rrBLUP and GCTA) to see the feasibility of the SDS method as a viable alternative to LMM-based methods. Our results (Table 1) show that the SDS method is inferior to the REML methods for small sample sizes, but it becomes progressively better and can match well with the precision of estimation by the REML methods for large sample sizes. Thus, these results suggest that the SDS method can serve as a viable alternative particularly when analyzing ‘big’ genomic data sets. Its major advantage is that with larger and larger data sets, it continues to work with the increasing precision of estimation while the other current commonly used methods are no longer able to work with our current computing capacity. To illustrate this point, we go beyond the parameter combinations set in Table 1 to simulate a larger data set with sample size of *n* = 50000 and marker density of *m* = 2000 and heritability of *h* ^2^ = 0.8. We analyze this data set using the SDS only because the other methods have already stopped to work with a sample of smaller size *n* = 40000 and the same marker density (*m* = 2000) as shown in Table 2. The means of estimates from 100 simulation samples are very similar and are closely around the true value of o.8 is 0.805 with SD being 0.026, and the 90% range is 0.072 (0.838–0.766). This range is much narrower than that for *n* = 5000. On average, the per-sample time requirement for constructing the GRM and the SDS analysis is 6355.3 seconds or about 1 hour 46 minutes.

It is evident from Table 2 that the SDS requires far less computational time and memory than the LMM methods. Henderson [9] and recently others [5]–[8], [12] have shown that the LMM estimates the variance components through simultaneously estimating the variance parameters and marker effects in an iterative manner. Usually, the iterative process starts with an initial (guessed) set of variance values or their ratio to provide the first round of estimates of random additive genetic effects (cf. equation A5 in the Text S1). These estimates of random effects are in turn used to estimates additive and residual variances using equation (A8) in the Text S1. The iteration continues until the successive rounds of estimates of variance components are stabilized. On the other hand, the SDS estimation of variance components or heritability is based directly on the linear regression of two sets second-order statistics, phenotypic and genetic similarities or dissimilarities. The SDS approach is much simpler and thus computationally much less demanding than the LMM approaches because it does not require (i) iteration and (ii) computing the inverse of GRM. The Bayesian analysis was not used in our study because in our initial investigation of different estimation methods, it took the Bayesian LASSO [17] 304.161 seconds or >5 minutes to complete the analysis of a single simulated sample of size *n* = 1000 with *m* = 2000, comparing to only 17.856 seconds by rrBLUP, 0.746 seconds by GCTA and 0.061 seconds by the SDS as shown in Table 2 for the same *n* and *m*. It would have been hardly feasible to run the data sets from a number of replicated simulations. Furthermore, de los Campos et al. [17] suggested the Bayesian analysis would be computationally even more demanding than the LMM analysis.

Computational efficiency has become an emerging issue particularly with increasing availability of larger and larger data sets from genomic studies and it has been recently investigated [8], [35]. However, these investigations focus on the computational complexity that is platform-independent, but ignore the implementation issues that may affect the actual computational efficiency in reality. In other words, the actual computational efficiency needs to be evaluated by considering the memory capacity and management under a given operating and software environment. Thus, in this study, we instead emphasize the *actual* computational time and memory requirements under different sample sizes (Table 2). Should the computational efficiency be based solely on the computational complexity [8], [35], the two REML implementations, rrBLUP and GCTA, would have had the same computational complexity. However, because rrBLUP is a cross-platform R package that is implemented strictly in the R environment and GCTA is a program that was written in C and has routinely run in the Linux operating system, the two programs are obviously different in terms of their actual computational efficiency (Table 2). This discrepancy is due mainly to the differences in the platforms and programming languages inherent in the two software packages.

In our study, any analysis would be terminated if its RAM usage exceeds 30GB, the maximum allowable RAMs after accounting for the RAM requirements by the operating system and other essential utility programs. It may be argued that we can increase the memory for the current computer or tap into supercomputing resources to address the issue of insufficient memory. However, neither solution is very feasible in our situation and perhaps in many other situations. Our workstation has a motherboard that can only support the maximum RAMs of 32GB and thus there is no room to add more memory. Many supercomputing servers such as the Westgrid in Canada (https://www.westgrid.ca/) have provided excellent computing resources to researchers with larger computing needs. However, access to these servers is a complicated process. It involves (i) submission of separate proposals for those systems with considerably large computing capacity in the supercomputing network; and (ii) a long waiting time after submitting batch jobs. In addition, some servers only serve certain countries or regions. Another possibility is the use of GPU (graphics processing unit)-accelerated computer for more efficient and even faster solutions to the large-scale GRM and its inverse essential in GWAS and genomic prediction. There are recent studies on the use of GPU-based parallel computing in GWAS [36]–[38], but these studies focus on detecting individual gene or gene-gene epistatic effects through genome-wide scan using single-marker analysis. Thus, it remains to be developed the GPU-based parallel computing algorithms for calculating the GRM and its inverse needed for genomic and marker-based estimation of variance components or heritability.

Our choice of rrBLUP and GCTA for comparison with the SDS method is somewhat arbitrary and it is due to our own familiarity and experiences with these software packages. Other LMM-REML software packages as mentioned in the Introduction section would have been equally effective for the comparison. ASREML [10] is widely used in agriculture community and it is implemented based on the average information algorithm as GCTA is. TASSEL [15] is very popular in plant breeding because it is able to accommodate both intra- and inter-population genetic relatedness in the LMM analysis. EMMA [6], [11] and its more efficient version GEMMA [8] have been extensively used in detection of causal variants responsible complex traits and diseases in human. FaST-LMM [12] uses a low-rank approximation to the GRM and thus it greatly improves computational efficiency and reduces memory requirement. For example, the computing time for the FaST-LMM analysis of a simulated data set with n = 20000 and m = 2000 is only 50.1 seconds and the maximum RAM usage for this run is 2.8 GB. These computational performance and memory requirement by FaST-LMM are much better than the GCTA analysis (1610.5 seconds and 29.0 GB), but are still not as good as the SDS analysis (2.9 seconds and 2.0 GB). Despite FaST-LMM’s superior computational performance and low memory requirement, it remains to be determined the optimal number of eigenvalues and corresponding eigenvectors that should be retained for a good low-rank approximation to the GRM for a given data set.

The analysis of the wheat yield data supplied by Crossa et al. [33] (Table 3) shows the similar estimates of heritability by the SDS and REML methods in all four environments, despite a relatively small sample size (*n* = 599) and low marker density (*m* = 1279). At the first glance, this result is somewhat surprising because the SDS estimates would have been more fluctuating than the REML at this level of sample size and marker density as can be inferred from the simulation results of Table 1. However, it should be remembered that this wheat population consists of 599 recombinant inbred lines (i.e., they are essentially 599 haplotypes) whereas our simulation data are all based on diploid individuals. The estimation of genetic relationship between haploids would be obviously more accurate than that between diploids at the same level of sample size and marker density. We also employ the GCTA analysis to confirm the estimates of genetic and residual variances as in Table 1 of Crossa et al. [33] based on their P model (i.e., the **A** matrix obtained from the pedigree information only) and the M-RKHS model (i.e., the **K** matrix obtained using the reproducing kernel Hilbert space nonlinear function of marker distance). The heritability estimates are subsequently calculated. Our GRM-based estimates of heritability, by either SDS or GCTA, are close to or slightly higher than those based on the P model, but they are all smaller than those based on the M-RKHS model. The elements of the K matrix derived from the M-RKHS model are a nonlinear (exponential) function of squared-Euclidean distance between markers in pairs of individuals, thereby probably capturing more genetic variation. It remains to be investigated how the use of nonlinear functions of marker distances generally affects the estimation of the genetic relationship between individuals, thereby influencing the estimation of variance components or heritability. The analysis of the drosophila data taken from Macdonald et al. [34] (Table 4) shows that the joint effect of all potential causal variants in in the E(spl)-C gene region remains small. This reinforces the earlier conclusion [34] that individual SNPs contribute little to the phenotypic variation in the bristle number.

A key part of both LMM and SDS analyses is to estimate the GRM based on a set of SNPs or other genetic markers. The use of GRM is more advantageous over the use of the traditional pedigree-based relationship matrix (**A** matrix) for two major reasons. First, the GRM would capture much of the Mendelian sampling variation that is missing in the **A** matrix. Second, the use of marker SNP data (rather than pedigree information) allows for estimation of relatedness of distantly related individuals, thereby controlling confounding effects from the environmental correlation between relatives due to their shared (common) environment. However, the unbiased estimate of GRM is achieved only if all the markers used for the estimation are the causal variants [4], [39], [40]. This of course is not true almost in all the cases. In our simulation studies, we use 10% of the total markers as the ‘causal’ (relevant) variants and the remaining 90% of the markers as irrelevant variants. It is evident from Table 1 that the accuracy of the estimated heritability is the best with the medium marker density (*m* = 2000) for most simulation trials. With the high marker density (*m* = 20000), there are more irrelevant markers, thereby costing some accuracy in the estimation of heritability. Similar results were observed in other simulation studies [e.g., 39].

In applying the SDS method to the actual analysis, we propose a modification to the Ritland’s estimation procedure. Instead of directly regressing the phenotypic similarity indexes on the corresponding genetic relatedness values between pairs of individuals as originally suggested in Ritland [26] and Lynch and Walsh [29, p. 800–803], we propose that the regression analysis should be based on the averages of bins to improve the feasibility and accuracy of the estimation in large genomic studies. The bins are constructed as a complete set of nonoverlapping intervals covering the entire range of genetic relatedness coefficients (i.e., all the elements of lower triangle of the GRM). The basis of our binning (grouping) procedure is simple: the neighboring values of genetic relatedness should contribute very similarly to the shape and pattern of the linear regression line in equation (9) and thus they can be bracketed into the same group or bin. A question would naturally arise: how many bins should we have to achieve the best estimates of variance components or heritability? In our own simulation and empirical data analysis, we use a set of 1000 equal-width bins for all the data. While this set is somewhat arbitrary, we feel it suffices for the data sets we have analyzed. Nevertheless, it is an interesting issue that needs to be further examined particularly when there are nonlinear relationships arising from gene-gene interactions and gene-environment interactions [3].

Another possible question with our binning procedure is that the number of observations may vary from bin to bin and thus the residual variance may also vary from bin to bin. At the first glimpse, the use of weighted least square (WLS) analysis would be a natural solution to this problem. However, our preliminary analysis shows that the WLS results are similar to those from the regression analysis of un-binned data (i.e., individual phenotypic and genetic similarities). We are not exactly sure why WLS does not work well as it should. We suspect the following reason. For the GRM constructed for a large sample, the distribution of genetic similarity between pairs of individuals is often highly skewed towards zero, that is, the majority of genetic similarity values are clustered around zero (see Figure S1 for an example of n = 4000 under the AR1 model with 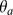 = 0.95 and *h* ^2^ = 0.5). In WLS, these near-zero values would be overemphasized (i.e., they would carry much more weight), thereby having a much stronger influence on the slope of the regression line (estimated additive genetic variance or narrow-sense heritability) than the values over the rest of the genetic similarity range. This issue certainly needs to be further investigated.

Our study focuses on a comparison of LMM-REML and SDS methods for estimating additive genetic variance and thus narrow-sense heritability. Such comparison can be easily extended to include non-additive genetic variances. As shown in Su et al. [41], the dominance genomic relationship matrix (**D**) based on marker genotypes is needed to estimate the dominance genetic variance; similarly different epistatic relationship matrices based on 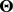 and **D** matrices (i.e., 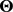
#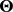, 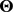
#**D**, **D**#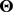 and **D**#**D**) are needed to estimate epistatic genetic variances. While it is quite straightforward for the SDS method to estimate all non-additive variances by extending the simple regression analysis to the multiple regression analysis, it is computationally even more challenging for the LMM-REML methods to estimate non-additive genetic variances even for a moderate-sized data set. Furthermore, far fewer software packages are available for the estimation of non-additive genetic variances. There is an ongoing debate on how much non-additive genetic variances really contribute to the ‘missing heritability’ [3], [42]–. According to Hill [42], such contribution would be relatively small for biologically more realistic epistatic models. Regardless, one of the key issues with the estimation of non-additive genetic variances is that unless sample size is large enough, the estimates would be highly unreliable. Thus, in the future efforts to improve the reliability of additive and non-additive variances from the analysis of large-scale genomic data sets, the LMM-REML methods will be certainly challenged but the SDS method looks very promising in terms of computational feasibility and estimation accuracy.

Our study uses all individuals, related and unrelated, in the simulated and empirical populations for the SDS and the LMM-REML analyses. This is somewhat in contrast to some recent studies, more specifically by Yang et al. [5] who focused on the estimation of additive genetic variance and narrow-sense heritability in a human population of ‘unrelated’ individuals with close relatives in their original population being selectively excluded from their LMM analysis. In particular, while the off-diagonal elements of the estimated GRM between 4259 individuals ranged from −0.024 to 0.585, Yang et al. [5] selectively chose individuals such that only those individuals with the genetic relatedness within the range of −0.024 to 0.024 were considered as ‘unrelated’ for the LMM-REML analysis. It is evident from Figure S1 that the majority of genetic similarity values for n = 5000 under the AR1 model and θ_a = 0.95 are clustered around zero with 95% of pairwise elements in the estimated GRM being from −0.0496 to 0.0524. This is slightly larger than but essentially similar to the range of −0.027 to 0.027 for the estimated GRM as given in Yang et al. [5]. It is also evident from Figure S1 that, of the remaining 5% elements in the estimated GRM under the AR1 model, the range from 0.0524 to 0.95 spans widely. It is this small percentage of the widespread values that largely determine the slope of the regression line in the SDS method. Similarly, we analyse the same data set using the usual LMM-REML analysis [e.g., 29] where all individuals are used regardless of their relatedness.

A distinction needs to be made between the use of simple regression analysis or its refinements for genome-wide scan of individual marker effects in a large-scale (*n* >100000) and *m* >500000) genomic studies [e.g., 23] and the use of LMM-REML analysis for the aggregate effect of all markers across the genome in our study and other studies. It should not be forgotten that a key motivation of using LMM-REML analysis is to help recover the portion of ‘missing’ heritability encountered in the single-marker analysis [5]. Earlier, we have already discussed some of the computational challenges with the LMM-REML analysis for a moderate-scale (*n* <10000) and *m* <100000) genomic data under our current typical computing capacity and suggested the SDS approach as a viable alternative when the data becomes larger and larger. With our binning approach to the SDS analysis, it is possible to handle genomic data sets of any size because the regression analysis is based on a fixed number of bins for phenotypic and genetic similarity indexes regardless of sample size. However, it is presently not feasible yet for the LMM-REML analysis to handle a very large data set. For example, Yang et al. [13] reported that it would take their software package GCTA ∼ 4 CPU hours (AMD Opteron 2.8 GHz) to compute the GRM for a data set with 3925 individuals genotyped by 294,831 SNPs. If we use this time as a guideline, then for a simulated data set with *m* = 500000 and *n* = 4000, it would have taken GCTA more than 400 ( = 100×4) CPU hours or more than 16 days to complete this simulation alone. Thus, in most recent LMM-REML analyses [e.g., 39], a moderate-sized data set is used.

## Conclusions

The SDS method that has been overshadowed by more popular LMM or Bayesian methods in recent years appears to have a bright future because of the computational challenges that the LMM and Bayesian methods are currently facing with growing availability of larger and larger genomic data sets from the genomic studies of human and domestic plants and animals. It can serve as a viable alternative framework for quantitative genomic analyses such as GWAS and genome-wide prediction. We hope that our study stimulates and renews research interests in the use of the SDS method for the analysis of large-scale genomic data sets that will become increasingly available in the future.

## Methods

### The SDS approach

There are two versions of the SDS approach. The first and true version of the SDS approach (SDS1) was originally proposed by Koch [45] and has been subsequently used in many genetics and genomics applications [3], [5], [24], [25], [30]. The second version that is based on the phenotypic covariance between pairs of related individuals (SDS2) was first put forward by Ritland [26] and subsequently discussed particularly in the genetic analysis of natural populations [27]–[29], [46]–[48]. However, the theoretical relationship between the versions has never been clarified so that they are sometimes considered as two different approaches [29]. In fact, we view them simply as the two sides of the same coin. In addition, there is a considerable amount of confusion regarding what needs to be used in the regression equation for estimation. Here we will describe both versions in details, point out the theoretical relationship between them and clarify appropriate and correct estimation procedures that should be used in each case.

#### SDS2

We describe the SDS2 first because it has a more direct connection with the LMM-REML analysis described in Text S1. Recall from the LMM analysis [equation (A2) in Text S1] that the total phenotypic covariance matrix among *n* individuals is partitioned into genetic and residual components, **V** = **G**+**R** = 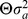
+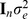, where 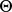 = {*θ_ij_*} is the *n*×*n* genetic relationship matrix (GRM) with *θ_ij_* being the coefficient of genetic relationship between *i*th and *j*th individuals, **I** *_n_* is an identity matrix order *n*, 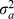 is the additive genetic variance and 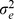 is the residual variance. Writing the phenotypic covariance between individuals *i* and *j* (*v_ij_*) in **V** in terms of the phenotypic correlation between the two individuals (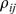) and their variances (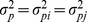), i.e., 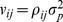, we have **V** = 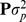 with **P** being the phenotypic correlation matrix and,
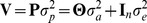
(1)


Multiplying both sides of equation (1) by 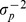, we obtain,
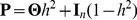
(2)where 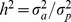 is the narrow-sense heritability. We now write the matrices in equations (1) and (2) in the vector form,
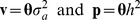
(3)where **v** = Vech(**V**), **θ** = Vech(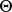), and **p** = Vech(**P**) with Vech(**X**) being the “vector-half” function [49] that creates a column vector whose elements are the stacked columns of the lower triangular elements of matrix **X**. Thus, for an *n*×*n* matrix **X**, there are *n*(*n*-1)/2 elements in Vech(**X**). The residual term in equation (3) disappears because Vech(**I** *_n_*) is a vector of zeros (**0**). The results in equation (3) reinforce the well-known result in classic quantitative genetics [29], [50] that for a quantitative trait with purely additive-genetic basis but no shared environmental effects, the phenotypic covariance between pairs of relatives in a population is expected to be the covariance between the additive genetic effects for the same pairs of relatives. Thus, equation (3) provides a simple regression model that can be used to estimate the variance components or heritability.

#### SDS1

Above we show that the linear relationship between phenotypic and additive genetic covariances or correlations between pairs of individuals can be used for predicting additive genetic variance or heritability. Similarly, the expected value of difference squared (DS) between the phenotypic values of a pair of individuals can be partitioned into the two components due to additive genetic effect and residual deviation as done in Grimes and Harvey [25]. Since the DS is symmetric for any pair of individuals (i.e., the DS is identical regardless of the order of the two individuals), Grimes and Harvey [25] called it the symmetric difference squared (SDS). This partitioning for the *i*th and *j*th individuals can be written as,
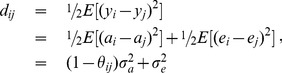
(4)where the quantity (1–*θ_ij_*) measures the genetic distance between the *ij*th pair of individuals for *i*<*j*. The usual assumptions are: (i) the additive genetic effects are independent of the residual deviations and (ii) there is no correlation between the residual deviations of the *ij*th pair of individuals. Collecting the partitioning results for all pairs of individuals including the trivial result of zero SDS for individuals with themselves, we have in matrix form,
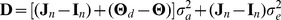
(5a)or
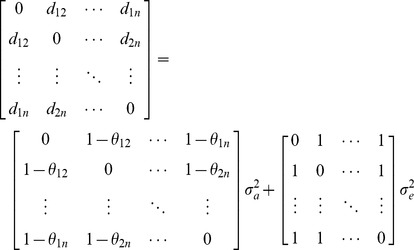
(5b)where **J** *_n_* is the *n*×*n* matrix of ones, **I** *_n_* is the identity matrix of order *n*, and 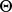 *_d_* is a diagonal matrix with the diagonal elements being the same as in matrix 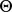.

#### Relationship between SDS1 and SDS2

The SDS1 and SDS2 (covariance) versions are obviously related to each other. This relationship between the phenotypic values of the *ij*th pair of individuals is derived as,
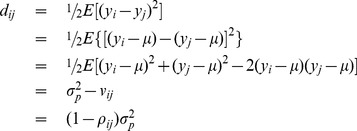
(6)


It is evident from equation (6) that a perfect inverse linear relationship between the phenotypic SDS (*d_ij_*) and the covariance (*v_ij_*) with the intercept being the phenotypic variance (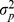) and the slope being −1. Thus, if the phenotypic SDS (*d_ij_*) values are known, we can immediately obtain the corresponding covariances for all pairs of individuals by reversing the relationship in equation (6) as *v_ij_* = 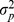 - *d_ij_*.

Similar relationships can be found for additive genetic SDS and residual SDS with 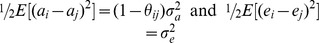.

Equation (4) shows the partitioning of the phenotypic SDS into additive genetic and residual variance components. To show the partitioning in terms of heritability, we need the standardized phenotypic SDS (*d_ij_*/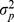) which is simply the phenotypic distance (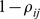). Thus, the standardized phenotypic SDS or phenotypic distance is related to the genetic distance and the heritability as,
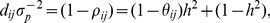
(7a)or in matrix form,
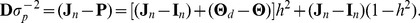
(7b)


#### Estimation procedure

It is evident from the above theoretical analysis that the estimation of variance components or heritability can be carried out using either the SDS1 (difference) model or SDS2 (covariance) model and both models would lead to the same estimates of variance components or heritability. Thus we will focus on our estimation procedure under the SDS2 model as it is more directly connected to the LMM analysis.

Before describing our own procedure, we outline the estimation procedure of Ritland [26] and Lynch and Walsh [29, p. 800–803] under the SDS2 model (also commonly known as Ritland’s procedure). For *n* individuals, there are *n*(*n*-1)/2 pairs of individuals and phenotypic values of each pair are used to calculate the phenotypic similarity. Thus, the sample phenotypic similarity between the *i*th and *j*th individuals for *i*<*j* is given by,
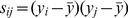
(8)where 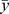 is the mean of *n* phenotypic values. Thus, the additive genetic variance can be estimated from the linear regression of the sample phenotypic similarity on the genetic relatedness as,
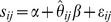
(9)where 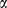 is the intercept, *β* is the regression coefficient which estimates the additive genetic variance (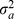), 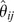 is the estimated value of 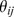 corresponding to the *ij*th element of the GRM estimated using *m* markers scored and 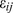 is the residual deviation of the sample phenotypic similarity from its expected value. The intercept should be zero because it is assumed that none of individual pairs are genetically identical, nor do they share the same environment. The estimate of additive genetic variance is simply the regression coefficient (slope),



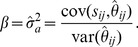
(10)The narrow-sense heritability is subsequently estimated as 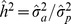. A more direct estimate of *h* ^2^ can be obtained by regressing the standardized phenotypic similarity index, 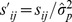, on the 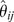 values,
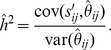
(11)


Ritland’s procedure has two major drawbacks particularly in the context of large-scale genomic studies. First, since it is not the sample phenotypic similarity index but rather its expected value that is proportional to the additive genetic variance or heritability without error[i.e., 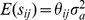 or 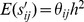], Ritland’s procedure makes the direct use of sample phenotypic similarity index and thus it would lead to a biased estimate of additive genetic variance or heritability [cf. equation (9)]. Second, while Ritland’s procedure is computationally much simpler than the above LMM analysis, it can still be memory- or time-consuming. For example, for 40000 individuals, the total pairs of phenotypic similarity values and genetic relatedness estimates are 799980000 and it certainly takes a long time and a large RAM capacity for the regression analysis based on such two huge arrays of values.

Here we propose a modification of Ritland’s procedure to remove the two deficiencies. Our new procedure is based on a simple idea that if we sort out the coefficients of genetic relatedness in an ascending or descending order, the neighboring values of genetic relatedness should contribute very similarly to the shape and pattern of the linear regression line in equation (9) and thus they can be bracketed into the same group or bin. Now the bin averages instead of individual phenotypic and genetic similarity indexes are used for the least squares estimation of additive genetic variance,
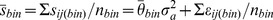
(12)


Clearly, the average of residual deviations (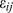
’s) would tend to zero for the large number of observations (i.e., large *n_bin_*) within the bins, thereby leading to the estimate of additive genetic variance with minimal bias. A similar argument can be made for binning the standardized phenotypic similarity index (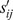) for estimating the heritability. In actual implementation of our binning strategy, we divide the whole range of genetic relatedness (usually 0–1) into 1000 equally spaced bins and then distribute individual similarity index values into different bins according to their levels of genetic relatedness. In other words, our binning approach uses the regression analysis based on this fixed number of bins regardless of the number of individual pairs and thus the sample size. Thus, its computing load is practically the same for samples of any sizes.

A similar estimation procedure can be given under the SDS1 model. The regression of the sample SDS (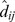) on estimated genetic relatedness (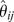) is given by,
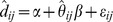
where the intercept *α* estimates the phenotypic variance (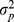 = 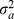
+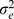) and the slope *β* estimates minus the additive genetic variance (-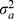) [cf. equation (4)]. In some studies [5], [24], twice the SDS value is used for the regression analysis and thus the intercept *α* estimates twice the phenotypic variance (2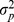) and the slope *β* estimates minus twice the additive genetic variance (−2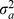).

### Simulation experiments

#### Simulation models and procedures

To investigate the dependence of phenotypic similarity on genetic relatedness, we need a population of individuals whose genetic relatedness covers the full range from no genetic correlation to perfect genetic correlation between pairs of individuals. Such special population is simulated using the first-order auto-regressive model (AR1). With a nearly perfect starting genetic correlation, AR1 model guarantees a full range of genetic relatedness for each and every simulated sample of sufficient size. Such high genetic correlation may be artificial and unrealistic for many human and livestock populations to which the LMM-REML analysis is often applied. However, it does approximate some situations in crop breeding where modern cultivars are genetically highly similar because (i) strong directional selection has been practiced and (ii) they all trace back to one or a few founders, for example, Canadian wheat [51].

We assume that the population consists of 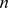 individuals which are arranged in a descending order according to the degrees of their genetic relatedness. Thus, the genetic correlation between any individual and its nearest neighbor is a constant 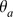, the genetic correlation between any individual and its second nearest neighbor (i.e., one individual apart) is 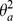, and so on. In general, the genetic correlation between the *i*th and *j*th individuals which are *t* = |*i* – *j*| individuals apart is 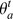. The correlation between *n* individuals under the AR1 model is,
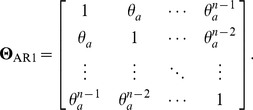
(13)


We further assume that 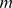 independent markers are genotyped for each individual. The simulation can be done by obtaining a 3×3 two-way contingency table for nine genotypes at a pair of SNP loci through sampling from a multinomial distribution. However, this procedure is time-consuming. We have found an equivalent but more efficient simulation procedure by directly sampling an *n*×*m* **Z** *^c^* matrix consisting of *m* random observations from an *n*-variate standard normal distribution with the mean vector of zeros and the covariance matrix being given in equation (13).

However, while this direct sampling scheme can be easily implemented using existing software packages such as the mvrnorm function in MASS/R [52] when 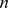 is not too large, it can be time-consuming for very large *n*. For this reason, a more efficient sampling scheme based on the definition of the AR1 model is employed and implemented in the following three steps. First, a 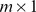 vector, 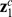, of random numbers are taken from the univariate standard normal distribution 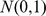 for the first individual. Second, another 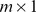 vector 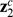 of random numbers are taken again from 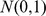 for the second individual given 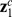, but with the correlation between 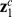 and 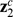 being 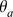. Thus a general recursive relationship for sampling the *i*th vector, 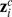 given the (*i*-1)th vector 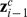 is
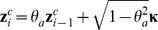
where 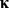 is a 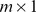 vector of random numbers that are taken from 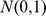. Third, all *n* **z** vectors generated in such way are collected to form the matrix **Z** ^c^ = 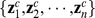.

Regardless of whether the **Z** ^c^ matrix is generated by directly sampling from a multivariate normal distribution or by the recursive relationship, it needs to be converted into an indicator genotype matrix **Z** = 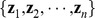 with vector **z** *_i_* containing only three values of 0, 1 and 2 to indicate three possible genotypes at each of *m* independent loci for the *i*th individual. For simplicity, we consider the proportions of the three genotypes in each individual to be 1∶ 2 : 1 so that the ranges for converting a normally distributed variate into the three genotypes coded as 0, 1 and 2 are: (−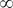,−0.67449), (−0.67449, 0.67449) and (0.67449, 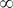), respectively. The genotypes could have been simulated directly through sampling from a multinomial distribution but the genetic relatedness between individuals are more conveniently accommodated through sampling from a multivariate normal distribution as in our simulation.

To simulate random additive genetic effects and phenotypic values, we assume that a quantitative trait is controlled by the quantitative trait loci (QTL) which are randomly located at one tenth of the total simulated markers (see Program S1 for step-by-step description of simulating positions and effects of causal markers). In other words, 10% of the total simulated markers are causal (relevant) variants and the remaining 90% are irrelevant markers. Thus a vector of phenotypic values (**y**) for 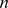 individuals are simulated using the following LMM model,
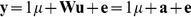
(14)where *µ* is the population mean, **W** is an *n*×*m* standardized genotype matrix with the *il*th element being 
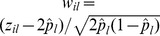
 and 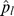 being the estimated frequency of the reference allele for the *l*th simulated marker, and **e** is a vector of *n* residual effects taken from a multivariate normal distribution, **u** is a vector of *m* random additive genetic effects that are taken from a standard multivariate normal distribution 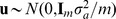 or equivalently the genome-wide additive genetic effects **a** = **Wu** are taken from a multivariate normal distribution, 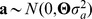 with 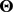 being estimated by 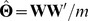, and 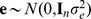. In our simulation, we have the phenotypic variance 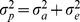 so that 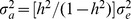 and 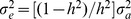 with 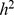 being the narrow-sense heritability.

#### Three simulation scenarios

We consider three simulation scenarios. In the first scenario, we investigate the degree of agreement between the actual and theoretical genetic relatedness. This scenario simulates five populations of sizes *n* = 200, 500, 1000, 2000 and 10000 with each population being genotyped at seven marker densities (*m* = 200, 500, 1000, 2000, 5000, 10000 and 20000). For each simulated population, the theoretical GRM between *n* individuals is obtained using the AR1 model as in equation (13). Here and throughout all simulations, we choose the genetic correlation between any individual and its nearest neighbor to be 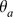 = 0.95. The actual GRM is estimated by 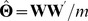 using each of the seven marker densities. The degree of agreement between the actual and theoretical genetic relatedness is measured by Pearson’s correlation between elements of matrices 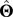 and 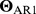.

In the second scenario, we want to examine the effects of sample size (*n*) and marker density (*m*) on the estimation of narrow-sense heritability. This simulation scenario consists of all combinations of the following parameter values: three levels of the heritability (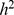 = 0.2, 0.5, and 0.8); three levels of sample size (*n* = 500, 1,000 and 5,000); and three levels of marker density (*m* = 200, 2,000 and 20,000). These simulation trials are replicated 100 times. The variance components and heritability are estimated using (i) the LMM approach as implemented by two software packages, rrBLUP [14] and GCTA [13], and (ii) the SDS approach as implemented by our own R package, SDS/R (http://statgen.ualberta.ca/index.html?open=software.html). In each of 100 replicated simulations, the LMM approach as in rrBLUP and GCTA estimates the additive genetic variance and residual variance and the heritability is subsequently calculated as the ratio of the estimated additive genetic variance to the sum of the estimated two variance components. On the other hand, the SDS approach directly estimates the heritability through the regression of the phenotypic correlation between pairs of individuals on the corresponding values of genetic relatedness.

In the third scenario, we want to compare and contrast the computational efficiency of the SDS method of heritability estimation to the REML-based methods as implemented in the two software packages, rrBLUP and GCTA over a wide range of sample sizes. We choose seven sample sizes for this scenario: *n* = 500, 1000, 2000, 5000, 10000, 20000, and 40000, but we only consider one marker density (*m* = 2000) and one heritability (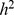 = 0.5). Given that the REML-based methods are very time-consuming for large *n*, each simulation trial is replicated only five times. The time (in seconds) required by the different estimation methods for the analysis of simulated data for each of the five replicates are recorded using proc.time, a R core function [53]. Since the GRM is required by all the estimation methods, the time needed for constructing the GRM is not included in the comparison of computational efficiency.

## Supporting Information

Figure S1 **The genetic similarity and phenotypic similarity in a simulated population under an AR1 model with** ***θ_a_*** **= 0.95 and** ***h*** **^2^ = 0.5, n = 4000 and m = 2000.**
(TIF)

Text S1 **Overview of linear mixed models for genomic data.**
(DOC)

Program S1 **R code for step-by-step description of simulating positions and effects of causal markers.**
(R) Click here for additional data file.
