# Supplementary figures and images for: Marker-Based Estimation of Genetic Parameters in Genomics
Source: PLoS One. 2014 Jul 15;9(7):e102715. doi: 10.1371/journal.pone.0102715 (PMC4099369; doi:10.1371/journal.pone.0102715)

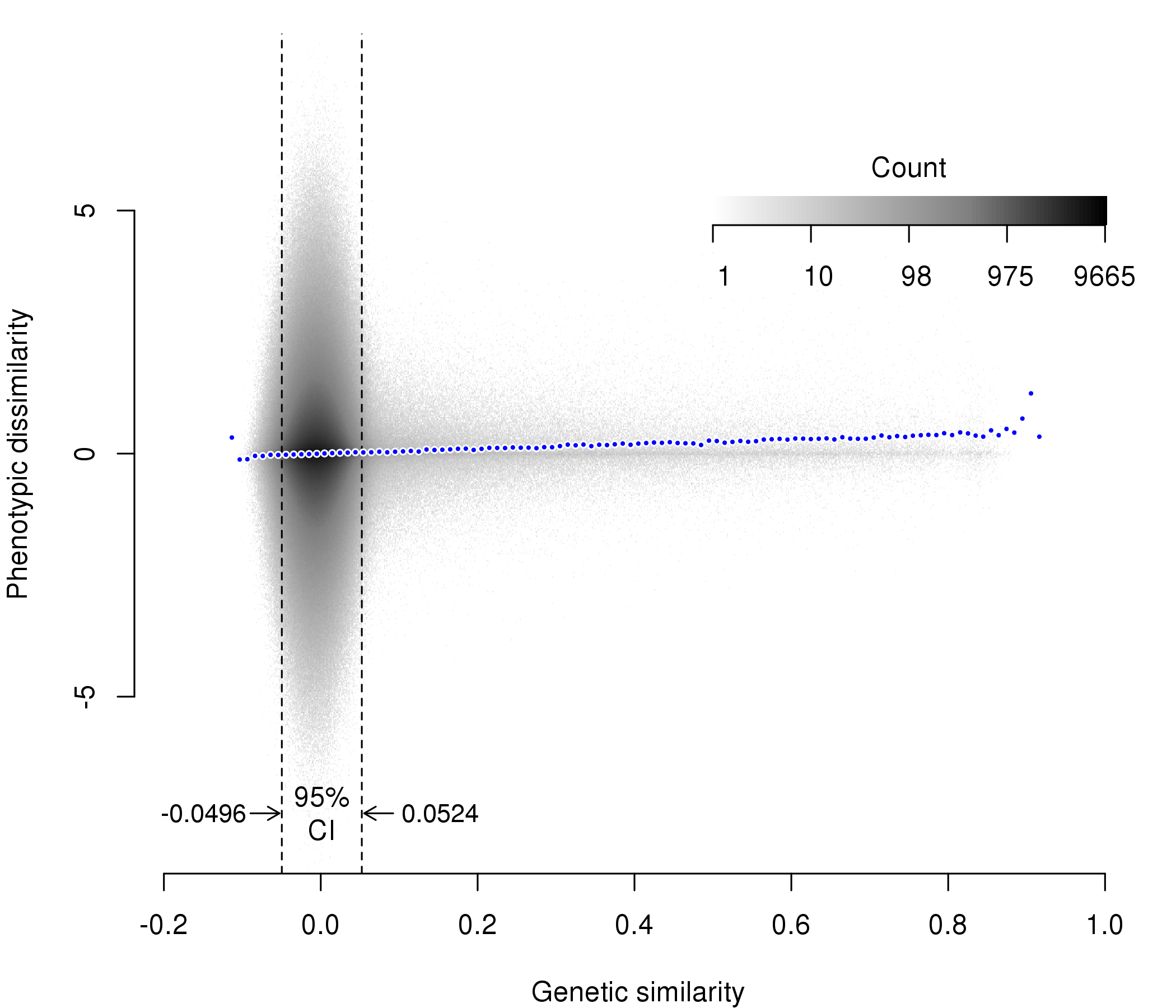

Supplement: Figure S1 — The genetic similarity and phenotypic similarity in a simulated population under an AR1 model with θa = 0.95 and h 2 = 0.5, n = 4000 and m = 2000. (TIF) [file pone.0102715.s001.tif]
