# Supplementary material for: Marker-Based Estimation of Genetic Parameters in Genomics
Source: PLoS One. 2014 Jul 15;9(7):e102715. doi: 10.1371/journal.pone.0102715 (PMC4099369; doi:10.1371/journal.pone.0102715)
Supplement: Text S1 — Overview of linear mixed models for genomic data. (DOC) [file pone.0102715.s002.doc]

### Additional file 1

# Overview of linear mixed models for genomic data

Here we will provide a brief overview of theory and procedures underlying the LMM-REML estimation of variance components and heritability. Such overview serves to demonstrate a natural connection with the SDS approach described in the **Methods** section.

The linear mixed model for fitting the effects of all the diallelic markers (e.g., SNPs) as random effects is,

$\mathbf{y}=\mathbf{X\beta}+\mathbf{Wu}+\mathbf{e}$ (A1)

where **y** is a vector of *n* phenotypic values, **β** is a vector of *k* fixed effects including the overall mean, **X** is an $n\times k$ design matrix that relates the phenotypes to the fixed effects, **u** is a vector of *m* marker random additive effects with $\mathbf{u}\sim N(\mathbf{0},\mathbf{I}_{m}\sigma_{u}^{2})$, **W** is an *n* × *m* mean-corrected or standardized genotype matrix with the *ij*th element being $w_{il}=z_{il}-2p_{l}$in the mean-corrected genotype matrix or $w_{il}=(z_{il}-2p_{l})/\sqrt{2p_{l}(1-p_{l})}$ in the standardized genotype matrix, where *z_il_* is the number of copies (0, 1 and 2) of the reference allele for the *l*th marker of the *i*th individual and *p_l_* is the frequency of the reference allele for the *l*th marker, and **e** is a vector of *n* residual effects with $\mathbf{e}\sim N(\mathbf{0},\mathbf{I}_{n}\sigma_{e}^{2})$. It is well known that the expected value and variance of vector **y** are E(**y**) = **Xβ** and Var(**y**) = **V** = **G** + **R**, where the additive genetic covariance matrix can be $\mathbf{G}=[\mathbf{WW}'/2\Sigma_{l}^{m}p_{l}(1-p_{l})]\sigma_{u}^{2}$if **W** is the mean-corrected genotype matrix or $\mathbf{G}=\mathbf{WW}'\sigma_{u}^{2}$ if **W** is the standardized genotype matrix, and the residual covariance matrix is $\mathbf{R}=\mathbf{I}_{n}\sigma_{e}^{2}$. Since the standardized genotype matrix is often used in the literature for constructing the **G** matrix, we will only use $\mathbf{G}=\mathbf{WW}'\sigma_{u}^{2}$ in our subsequent development and discussion.

Model (A1) is equivalent to the conventional mixed model with single record per individual,

$\mathbf{y}=\mathbf{X\beta}+\mathbf{a}+\mathbf{e}$ (A2)

if Var(**a**) = $\mathbf{G}=\mathbf{\Theta}\sigma_{a}^{2}$= $\mathbf{WW}'\sigma_{u}^{2}$, where **a** = **Wu** is an *n* × 1 vector of the total additive genetic effects for the *n* individuals with $\mathbf{a}\sim N(\mathbf{0},\mathbf{\Theta}\sigma_{a}^{2})$ and $\mathbf{\Theta}$being the *n* × *n* genetic relationship matrix (GRM) between individuals. In the past, the GRM has been estimated using known pedigrees among individuals (i.e., **A** matrix), with the *ij*th element of the GRM being for diploids or for haploids, where is the kinship coefficient between the *i*th and *j*th individuals . Now the routine use of marker genotypes for estimating the GRM allows for capturing additional genetic variation due to Mendelian sampling . However, a marker-based estimate of the GRM is unbiased only if it is based on the QTL or causal variants .

The (*k* + *m*) mixed-model equations (MMEs) for **β** and **u** in model (A1) can be solved to obtain the best linear unbiased estimation (BLUE) of fixed effects **β** and the best linear unbiased prediction (BLUP) of random effects **u**,

$\left[\begin{matrix} \hat{\mathbf{\beta}} \\ \hat{\mathbf{u}} \end{matrix}\right]=\left[\begin{matrix} \mathbf{X}'\mathbf{R}^{-1}\mathbf{X} & \mathbf{X}'\mathbf{R}^{-1}\mathbf{W} \\ \mathbf{W}'\mathbf{R}^{-1}\mathbf{X} & \mathbf{W}'\mathbf{R}^{-1}\mathbf{W}+\mathbf{G}^{-1} \end{matrix}\right]^{-}\left[\begin{matrix} \mathbf{X}'\mathbf{R}^{-1}\mathbf{y} \\ \mathbf{W}'\mathbf{R}^{-1}\mathbf{y} \end{matrix}\right]$ (A3)

with superscript minus one (^-1^) and superscript minus (^-^) representing matrix and generalized inverses, respectively, and $\mathbf{G}^{-1}=\mathbf{I}_{m}\sigma_{u}^{-2}$. The coefficient matrix in equation (A3) is also known as the **C** matrix ,

$\mathbf{C}=\left[\begin{matrix} \mathbf{C}_{\mathbf{\beta\beta}} & \mathbf{C}'_{\mathbf{u\beta}} \\ \mathbf{C}_{\mathbf{u\beta}} & \mathbf{C}_{\mathbf{uu}} \end{matrix}\right]=\left[\begin{matrix} \mathbf{X}'\mathbf{R}^{-1}\mathbf{X} & \mathbf{X}'\mathbf{R}^{-1}\mathbf{W} \\ \mathbf{W}'\mathbf{R}^{-1}\mathbf{X} & \mathbf{W}'\mathbf{R}^{-1}\mathbf{W}+\mathbf{G}^{-1} \end{matrix}\right]^{-}$ (A4)

where

$\begin{matrix} \mathbf{C}_{\mathbf{\beta\beta}}=\mathbf{(X'V}^{-\mathbf{1}}\mathbf{X)}^{-} \\ \mathbf{C}_{\mathbf{u\beta}}=-\mathbf{GW'V}^{-\mathbf{1}}\mathbf{XC}_{\mathbf{\beta\beta}} \\ \mathbf{C}_{\mathbf{uu}}=\mathbf{(W'R}^{-\mathbf{1}}\mathbf{W}+\mathbf{G}^{-\mathbf{1}}\mathbf{)}^{-\mathbf{1}}-\mathbf{C}_{\mathbf{u\beta}}\mathbf{X'V}^{-\mathbf{1}}\mathbf{WG} \end{matrix}$

Similarly, the (*k* + *n*) MMEs for **β** and **a** in model (A2) is given by,

$\left[\begin{matrix} \hat{\mathbf{\beta}} \\ \hat{\mathbf{a}} \end{matrix}\right]=\left[\begin{matrix} \mathbf{X}'\mathbf{R}^{-1}\mathbf{X} & \mathbf{X}'\mathbf{R}^{-1} \\ \mathbf{R}^{-1}\mathbf{X} & \mathbf{R}^{-1}+\mathbf{G}^{-1} \end{matrix}\right]^{-}\left[\begin{matrix} \mathbf{X}'\mathbf{R}^{-1}\mathbf{y} \\ \mathbf{R}^{-1}\mathbf{y} \end{matrix}\right]$ (A5)

and the **C** matrix can be written as,

$\mathbf{C}=\left[\begin{matrix} \mathbf{C}_{\mathbf{\beta\beta}} & \mathbf{C}'_{\mathbf{u\beta}} \\ \mathbf{C}_{\mathbf{u\beta}} & \mathbf{C}_{\mathbf{uu}} \end{matrix}\right]=\left[\begin{matrix} \mathbf{X}'\mathbf{R}^{-1}\mathbf{X} & \mathbf{X}'\mathbf{R}^{-1} \\ \mathbf{R}^{-1}\mathbf{X} & \mathbf{R}^{-1}+\mathbf{G}^{-1} \end{matrix}\right]^{-}$ (A6)

where

$\begin{matrix} \mathbf{C}_{\mathbf{\beta\beta}}=\mathbf{(X'V}^{-\mathbf{1}}\mathbf{X)}^{-} \\ \mathbf{C}_{\mathbf{u\beta}}=-\mathbf{GV}^{-\mathbf{1}}\mathbf{XC}_{\mathbf{\beta\beta}} \\ \mathbf{C}_{\mathbf{uu}}=\mathbf{(R}^{-\mathbf{1}}+\mathbf{G}^{-\mathbf{1}}\mathbf{)}^{-\mathbf{1}}-\mathbf{C}_{\mathbf{u\beta}}\mathbf{X'V}^{-\mathbf{1}}\mathbf{G} \end{matrix}$

It should be noted that $\mathbf{G}^{-1}=\mathbf{\Theta}^{-1}\sigma_{a}^{-2}$exists only if $\mathbf{\Theta}$ is positive definite and thus investable. With high marker densities, the direct use of MMEs for obtaining the BLUP of marker effects **u** under model (A1) may become computationally challenging. In this case, model (A2) is used to predict the genetic effects **a** first and then the marker effects **u** is obtainable as ,

$\hat{\mathbf{u}}=\mathbf{W}'\mathbf{\Theta}^{-1}\hat{\mathbf{a}}$. (A7)

REML estimators of variance components can be obtained by several algorithms including derivative-based methods such as the Newton-Raphson algorithm and Fisher’s scoring method, EM (expectation-maximization) methods and Average Information (AI) algorithm . All these methods are computationally intensive. For example, the REML estimators of genetic variance ($\sigma_{a}^{2}$) and residual variance ($\sigma_{e}^{2}$) through the EM algorithm requires iterating on,

$\begin{matrix} \hat{\sigma}_{e}^{2}=(\mathbf{y}'\mathbf{R}^{-1}\mathbf{y}-\hat{\mathbf{\beta}}'\mathbf{X}'\mathbf{R}^{-1}\mathbf{y}-\hat{\mathbf{a}}'\mathbf{R}^{-1}\mathbf{y})/[n-r(\mathbf{X})] \\ \hat{\sigma}_{a}^{2}=[\hat{\mathbf{a}}'\mathbf{\Theta}^{-1}\hat{\mathbf{a}}+\hat{\sigma}_{e}^{2}\text{tr}(\mathbf{\Theta}^{-1}\mathbf{C}_{\mathbf{uu}})]/n \end{matrix}$ (A8)

where *r*(**X**) is the rank of matrix **X** and tr() stands for the trace of the matrix. The solutions require computing $\mathbf{\Theta}^{-1}$ and **C_uu_** . Both of these matrices are difficult to compute when *n* is large. In implementing the AI algorithm, some of the mixed model analysis packages, such as ASREML , have avoided the inversion of the *n* × *n* **V** matrix using the Gaussian elimination of the MME to obtain the AI matrix based on sparse matrix techniques. However, as [Yang, et al. [13]](#_ENREF_13) pointed out, since the marker-based GRM matrix is usually dense, the use of the sparse matrix technique for the GRM matrix will actually lead to an extra cost of memory and CPU time. In general, the relative performance of different computing strategies is dependent on the number of individuals, the number of marker loci per individual, and the number of iterations required to solve the MMEs .

# References

1. Lynch M, Walsh B: *Genetics and analysis of quantitative traits.* Sunderland, MA, USA: Sinauer Associates; 1998.

2. Henderson CR: *Applications of Linear Models in Animal Breeding.* University of Guelph; 1984.

3. Falconer DS, Mackay TFC: *Introduction to quantitative genetics.* 4th edn: Longman New York; 1996.

4. VanRaden PM: **Efficient methods to compute genomic predictions.** *J Dairy Sci* 2008, **91:**4414-4423.

5. Yang J, Benyamin B, McEvoy BP, Gordon S, Henders AK, Nyholt DR, Madden PA, Heath AC, Martin NG, Montgomery GW, Goddard ME, Visscher PM: **Common SNPs explain a large proportion of the heritability for human height.** *Nat Genet* 2010, **42:**565-569.

6. Goddard ME, Hayes BJ, Meuwissen THE: **Using the genomic relationship matrix to predict the accuracy of genomic selection.** *J Anim Breed Genet* 2011, **128:**409-421.

7. Lippert C, Quon G, Kang EY, Kadie CM, Listgarten J, Heckerman D: **The benefits of selecting phenotype-specific variants for applications of mixed models in genomics.** *Scientific Reports* 2013, **3:**1815.

8. Zaitlen N, Kraft P: **Heritability in the genome-wide association era.** *Hum Genet* 2012, **131:**1655-1664.

9. Mclean RA, Sanders WL, Stroup WW: **A unified approach to mixed linear-models.** *Am Stat* 1991, **45:**54-64.

10. Yang R-C: **Towards understanding and use of mixed-model analysis of agricultural experiments.** *Can J Plant Sci* 2010, **90:**605-627.

11. Henderson CR: **Mivque and Reml Estimation of Additive and Nonadditive Genetic Variances.** *J Anim Sci* 1985, **61:**113-121.

12. Piepho HP, Ogutu JO, Schulz-Streeck T, Estaghvirou B, Gordillo A, Technow F: **Efficient Computation of Ridge-Regression Best Linear Unbiased Prediction in Genomic Selection in Plant Breeding.** *Crop Sci* 2012, **52:**1093-1104.

13. Yang JA, Lee SH, Goddard ME, Visscher PM: **GCTA: A tool for genome-wide complex trait analysis.** *Am J Hum Genet* 2011, **88:**76-82.

14. Gilmour AR, Gogel B, Cullis B, Thompson R: **ASReml user guide release 3.0.** *VSN International Ltd, Hemel Hempstead, UK* 2009.

15. Stranden I, Garrick DJ: **Derivation of equivalent computing algorithms for genomic predictions and reliabilities of animal merit.** *J Dairy Sci* 2009, **92:**2971-2975.
